# The Pivotal Interaction Between Serotonin and Calcium Shifts in Lactating Pregnant Spanish Purebred Mares: The Aging Effect

**DOI:** 10.3390/vetsci12050398

**Published:** 2025-04-23

**Authors:** Katiuska Satué, Esterina Fazio, Pietro Medica, Maria Gemma Velasco-Martinez, Cristina Cravana, Giuseppe Bruschetta, Deborah La Fauci

**Affiliations:** 1 Department of Animal Medicine and Surgery, Faculty of Veterinary, Cardenal Herrera-CEU University, 46115 Valencia, Spain; ksatue@uchceu.es (K.S.); mariagemma.velasco@alumnos.uchceu.es (M.G.V.-M.); 2 Unit of Veterinary Physiology, Department of Veterinary Sciences, Messina University, Polo Universitario Annunziata, 98168 Messina, Italy; fazio@unime.it (E.F.); ccravana@unime.it (C.C.); gbruschetta@unime.it (G.B.); deborah.lafauci@unime.it (D.L.F.)

**Keywords:** serotonin, ionized/total calcium, mares, pregnancy, lactation

## Abstract

During the pregnancy and lactation phases, physiological adaptations occur in the mother to cope with the additional nutritional demands of the fetus and newborn. In experimental animals and cows, serotonin (5-HT) induces augmented bone mobilization by increasing calcium (Ca^2+^) concentrations in blood and milk during pregnancy and/or lactation. These interactions between 5-HT and Ca^2+^ homeostasis remain unknown in mares. Hence, the hypothesis of this study was that, as in other species, mares’ 5-HT and Ca^2+^ concentrations are influenced by pregnancy and lactation and that this relationship could be influenced by age. The aim was to verify the existence of a bidirectional interaction between circulating 5-HT and ionized (ICa^2+^) and total (TCa^2+^) Ca^2+^ shifts in thirty-one healthy lactating Spanish Purebred mares during pregnancy, evaluating the effect of different ages (<10- and >10 years old). Compared to >10-year-old mares, those aged <10 years old showed a greater 5-HT concentration from the 3rd to the 8th month of pregnancy (*p* < 0.05), a greater ICa^2+^ concentration from the 5th to the 8th month (*p* < 0.05), a lower TCa^2+^ concentration from the 1st to the 3rd month (*p* < 0.05), a greater concentration at the 7th, 8th, and 11th month (*p* < 0.05), and a greater ICa^2+^/TCa^2+^ ratio from the 5th to the 7th month (*p* < 0.05). The data obtained show an interesting and significant relationship between circulating 5-HT and both ICa^2+^ and TCa^2+^, as well as between ICa^2+^ and TCa^2+^. Moreover, aging appears to reduce the secretory tone of 5-HT, with a concurrent large shift in Ca^2+^ metabolism in lactating pregnant mares.

## Simple Summary

This is an experimental study carried out on thirty-one healthy lactating and pregnant Spanish Purebred mares to evaluate if, in equines, like other species, serotonin (5-HT) induces increased bone mobilization by augmenting calcium (Ca^2+^) concentrations in blood and milk during pregnancy and/or lactation. The hypothesis of this study was that plasma 5-HT and ionized (ICa^2+^) and total Ca^2+^ (TCa^2+^) could vary under pregnancy and lactation conditions in mares of advancing age, providing information not only from a physiological but also from a clinical point of view in the equine species. Positive relationships between circulating 5-HT and both ICa^2+^ and TCa^2+^, as well as between ICa^2+^ and TCa^2+^, were observed. Aging appears to reduce the secretory tone of 5-HT, with a concurrent large shift in Ca^2+^ metabolism in lactating pregnant mares.

## 1. Introduction

During pregnancy, circulating serotonin or 5-hydroxytryptamine (5-HT) is involved in critical physiological functions such as the mobilization of energy sources throughout embryogenesis, morphogenesis, cell differentiation, and proliferation [1]. 5-HT regulates human vascular tone, modulates placental functionality and fetal development [2], and stimulates pancreatic β cell proliferation with successive insulin release, modulating the nutrient supply for fetal growth in experimental animals [3]. In pregnant mares, the activation of the sympathetic nervous system (SNS) during successful pregnancies was recorded, with a significant increase in plasma 5-HT in the 5th and 7th month and a decrease during the last three months of pregnancy, with the lowest values at the 11th month [4]; however, its effects are not yet fully known.

In the last decade, particular attention has been directed toward better clarifying the physiological role of calcium (Ca^2+^) metabolism [5,6] and related changes in dairy cattle, with more emphasis on the transition and lactation period [7]. 5-HT regulates Ca^2+^ dynamics during lactation, being a vital tool in rodent models and in lactating cows, in which 5-HT was positively correlated with circulating total Ca^2+^ (TCa^2+^) on the first day of lactation [7]. 5-HT induces increased bone mobilization in cows by stimulating the parathyroid hormone and bone resorption in osteoclasts and increasing Ca^2+^ concentrations in milk [8], as occurs in the blood of experimental animals during pregnancy [9].

Profiles of TCa^2+^ concentrations both in cyclic and pregnant mares have been well documented. Indeed, serum Ca^2+^ concentrations decreased slightly during lactation and the first 2 months of pregnancy when some mares were in the lactation phase compared to the concentrations observed mid-pregnancy [10]. However, no significant difference in serum Ca^2+^ concentration was reported in the Thoroughbred pregnant versus nonpregnant mares by Holley and Evans (1977) [11]. In addition, it is well known that mares at 2 post-partum days show a transient 12% decrease in circulating Ca^2+^ concentration associated with a transient three-fold increase in parathyroid hormone concentration [12]. Moreover, serum Ca^2+^ significantly increases at 20 ± 10 and 60 ± 10 days after foaling compared to 60 ± 10 days before parturition [13]. In mares, peak lactation occurs between 20 and 90 days according to different breeds; in fact, heavy breeds have a later peak than saddle horses, with greater production and a delayed peak in breeds characterized by a greater growth rate in the first months of life [14]. It is, therefore, feasible that aging represents a gradual but irreversible physio-pathological process involving declines both in tissue and cell functions [15,16,17]. However, free Ca^2+^ or ionized (ICa^2+^) is more useful than TCa^2+^ and provides the best indication of calcemic status since ICa^2+^ is biologically active and closely regulated by Ca^2+^-modulating hormones [18]. To the authors’ knowledge, ICa^2+^ concentrations have been only assessed in pregnant Thoroughbred and Quarter horse mares during the third trimester of pregnancy [18] and the post-partum period [10] or in those suffering from colic episodes during this period [19].

Recently, in nonpregnant mares, these same researchers showed that advancing age leads to a predominance of the SNS and a profound drop in serotonergic activity, with lower 5-HT concentrations in mares aged > 16 years old versus those aged 6–9, 10–12, and 13–16 years [20]. Although it is not known in mares, in women, it is well-documented that alterations in serum 5-HT concentrations during gestation are associated with hypertension, increased risk of premature births, low birth weight, and neonatal neurological disorders [21]. In the same way, dysregulation of 5-HT during the peri-partum period leads to hypocalcemia in cows. Although post-partum hypocalcemia is very common in high-production cattle, it is rare in horses [22,23].

On this basis, the existence of a dynamic 5-HT–calcium axis during the evolution of normal fetal development processes along with successful pregnancy and lactation in the mare should be clearly established by also assuming the aging effect. The hypothesis of this study was that circulating 5-HT, ICa^2+^, and TCa^2+^ could vary under pregnancy and lactation phases in mares of advancing age, providing information not only from a physiological but also from a clinical point of view. The current research aims to verify the existence of a bidirectional interaction between 5-HT and Ca^2+^ shifts in lactating pregnant Spanish Purebred mares, evaluating the effect of aging.

## 2. Materials and Methods

### 2.1. Mares

This study included thirty-one healthy reproductive Spanish Purebred mares stabled on different farms located in the same geographic area (Valencian Community, longitude: 0°45′0″ W, and latitude: 39°30′0″ N) and classified according to age into two groups, consisting of 15 subjects < 10 years old (from 6 to 9 years) and 16 subjects > 10 years old (from 10 to 15 years). The inclusion criteria were the following: (i) to be healthy mares without reproductive disorders or disease; (ii) to be properly dewormed and vaccinated without any medical or surgical treatment in the three months before the start of the study; (iii) to have become pregnant between mid-February and mid-March; and (iv) to have obtained written consent from the owners.

All mares received the same routine handling protocols and feeding regimes. During the day, they were housed in groups outdoors in large paddocks and subsequently collected at night, and in periods of inclement weather, they were housed in individual stalls. Mares were fed alfalfa hay and mixed grains, represented mainly by oats and small amounts of barley, and access to water was ad libitum. The mares’ insemination was performed using frozen semen from the stallions of the farms.

The mares were intended for breeding, this being the only activity carried out from the age of 4 years, and in no case were they subjected to training or regulated physical exercise. Of the total 11 months of gestation, all mares had their foals at their feet and were in the lactation period for the first 5 months. The foals were born healthy and did not present any clinical incidence during the study period. Since the study began with gestation immediately after birth, the mothers were lactating, and all the foals were 1 month old and were thereafter weaned at the age of 5 months.

During the sampling period, the composition of the diet of the mares consisted of a combination of fiber and compounded food divided into two meals a day; the fiber included 2–3 kg of alfalfa hay and straw, with a natural calcium content of 15 g (composition/kg of food) or 18.75 g (administered/100 Kg BW). Specifically, mares during the first 8 months of gestation were fed hay ad libitum (equivalent to approximately 7.5 kg) and 4 kg of concentrated feed based on barley, oats, corn, and wheat. Subsequently, from this period until delivery, their diet was supplemented using a special feed (Pavo^®^, Pinto, Madrid, Spain) at a dose of 0.42 kg per 100 kg of live weight per day. Water was given ad libitum.

### 2.2. Blood Samples and Analyses

Blood samples were taken from the jugular veins of thirty-one healthy Spanish Purebred mares for the entire pregnancy period, with monthly intervals of 30 days between each sampling. This interval period was controlled individually for each mare according to the date of insemination. Blood samples started 16 days after insemination, corresponding to the onset of pregnancy, which was confirmed by ultrasound. All mares enrolled in this study became pregnant between mid-February and mid-March. The last blood sample was taken between 7 and 15 days before the date of delivery. In order to not be affected by the circadian effect on hormonal patterns, blood samples were taken in the morning between 08:00 and 10:00, at rest, and always by the same operator. Immediately after sampling, the blood was transferred to heparinized tubes (Tapval^®^, Barcelona, Spain). After 30 min, the samples were centrifuged at 3000× *g* for 10 min at 4 °C, and the plasma was harvested and stored at −80 °C until analysis.

### 2.3. Analytical Procedures

5-HT (mg/dL) concentrations were analyzed by an enzyme-linked immunosorbent assay (ELISA) (DLD Diagnostike GmbH). This technique shows the cross-reactivities of the polyclonal antibody with 5-HT (100%), tryptamine (1.3%), 5-methoxytryptamine (0.18%), and melatonin (<0.013%) and has been validated and used previously for horses [24].

Ionized Ca^2+^ (ICa^2+^) concentrations were analyzed in heparinized samples (95 µL of blood with 150 IU heparin/mL) by an i-STAT Alinity veterinary blood analyzer using i-STAT CHEM8+ Cartridge (REF 09P31-25) (Abbot, U.S.) according to the manufacturer’s instructions. The principle of the technique is based on an ion-selective electrode potentiometer, and i-STAT’s Advanced Quality Functions (AQFs) helped optimally manage a point-of-care (POC) testing program to improve the analyzer’s compliance, monitoring, and control. The coefficients of variation of the technique were <2.5%.

Total Ca^2+^ (TCa^2+^) was determined by spectrophotometry using reagents from the same company (Spinreact 200; Tarragona, Spain). The detection and linearity limits were 0.17 mg/dL and 15 mg/dL, respectively. The intra- and inter-assay coefficients of variation ranged from 0.68 to 0.81 and 1.76 to 1.89, respectively. The ratio between ICa^2+^ and T Ca^2+^ (ICa^2+^/TCa^2+^) was also calculated.

### 2.4. Statistical Analyses

Statistical analysis was carried out using the program SPSS 12.01 for Windows (SPSS Inc., Chicago, IL, USA). A multivariate regression analysis with repeated measurements to analyze the variations experienced by 5-HT, ICa^2+^, TCa^2+^, and the ratio of Ca^2+^/TCa^2+^during lactation period was realized. The relationship between these parameters was examined by linear regression analysis, and the correlation was expressed by Pearson’s correlation coefficient. The timepoint in lactation was included in the regression analysis. Differences were statistically significant when *p* < 0.05.

## 3. Results

**Figure 1:**
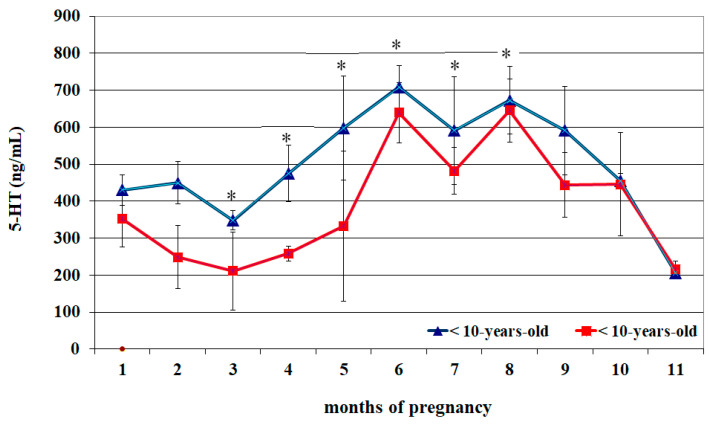
Circulating serotonin (5-HT) concentrations (Mean ± S.D.) in lactating pregnant Spanish Purebred mares aged < 10- and >10 years old throughout the entire pregnancy. Asterisk indicates significant differences versus >10-year-old mares (*p* < 0.05).

Circulating 5-HT profiles of <10- and >10-year-old mares appeared almost superimposable (Figure 1) and were characterized by lower concentrations from the 1st to the 3rd month of pregnancy than the 5th–10th month (*p* < 0.05). An increase in concentrations was observed from the 4th month to the 6th month, whose peak was greater than the previous two months (*p* < 0.05), followed by a progressive decrease and reaching lower concentrations at the 11th month than all previous months (*p* < 0.05) (Table 1). Compared to >10-year-old mares, those aged < 10 years old showed greater 5-HT concentrations from the 3rd to the 8th month of pregnancy (*p* < 0.05) (Figure 1).

The circulating ICa^2+^ of <10-year-old mares showed lower concentrations from the 1st to the 4th month of pregnancy and again at the 11th month (*p* < 0.05) than the values recorded from the 5th to the 9th month (*p* < 0.05), followed by greater values at the 5th and 6th month (*p* < 0.05) than at the 8th–11th month and with a progressive decrease until the 11th month (Table 2).

The circulating ICa^2+^ of >10-year-old mares showed lower concentrations from the 1st to the 4th month of pregnancy and again at the 11th month (*p* < 0.05) than the values recorded at the 7th and 9th month (*p* < 0.05) (Table 2).

**Figure 2:**
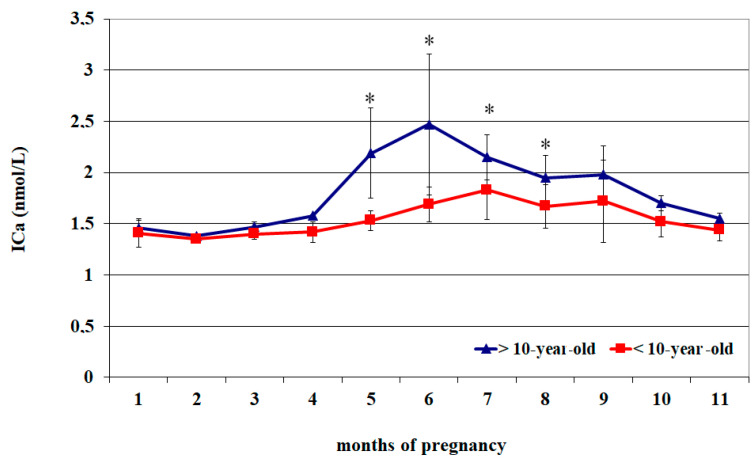
Circulating ionized calcium (ICa^2^) concentrations (Mean ± S.D.) in lactating pregnant Spanish Purebred mares aged < 10- and >10 years old throughout the entire pregnancy. Asterisk indicates significant differences versus >10-year-old mares (*p* < 0.05).

Compared to >10-year-old mares, those aged < 10 years old showed greater ICa^2+^ concentrations from the 5th to the 8th month of pregnancy (*p* < 0.05) (Figure 2).

**Figure 3:**
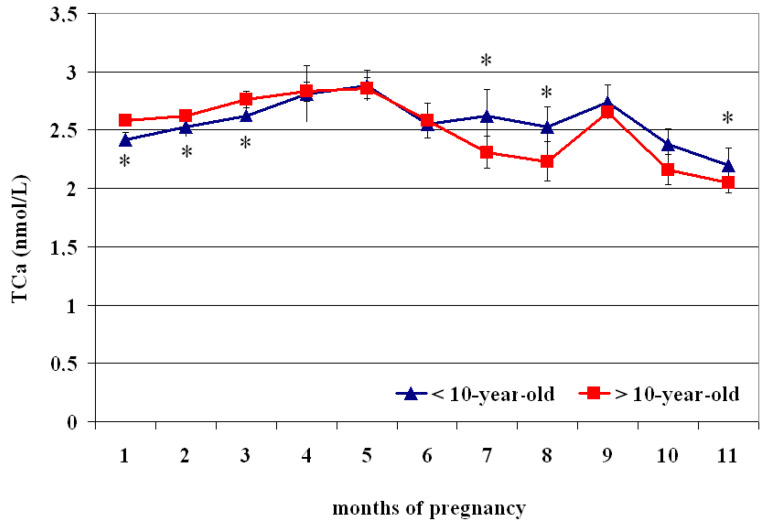
Circulating total calcium (TCa^2+^) concentrations (Mean ± S.D.) in lactating pregnant Spanish Purebred mares aged < 10- and >10 years old throughout the entire pregnancy. Asterisk indicates significant differences versus >10-year-old mares (*p* < 0.05).

The circulating TCa^2+^ concentrations showed superimposed profiles both in <10- and >10 year olds (Figure 3), with lower values at the 1st–2nd month than the 3rd–5th month (*p* < 0.05) and greater at the 4th–5th month than the 6th–11th month (*p* < 0.05); greater values were recorded at the 9th month than the 10th–11th month (*p* < 0.05) (Table 3). Compared to >10 year olds, those aged < 10 showed lower TCa^2+^ concentrations from the 1st to the 3rd month and greater values at the 7th, 8th, and 11th month of pregnancy (*p* < 0.05) (Figure 3).

**Figure 4:**
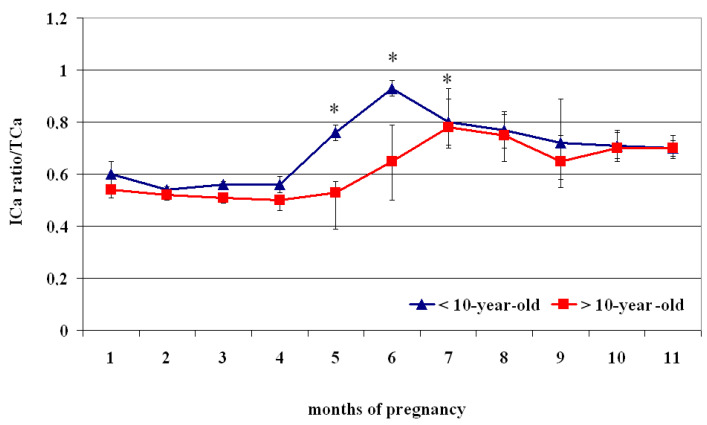
Ionized calcium (ICa^2+^) and total calcium (TCa^2+^) ratio (Mean ± S.D.) in lactating pregnant Spanish Purebred mares aged < 10- and >10 years old throughout the entire pregnancy. Asterisk indicates significant differences vs. >10-year-old mares (*p* < 0.05).

The ICa^2+^/TCa^2+^Ca ratio showed a biphasic profile, with lower values from the 1st to the 4th month than the following periods (*p* < 0.05) (Figure 4, Table 4). Specifically, <10-year-old mares showed lower concentrations at the 1st month of pregnancy than at the 5th–9th month (*p* < 0.05), lower values from the 2nd to the 4th month than those recorded at the 5th–11th month (*p* < 0.05), and lower values at the 5th month than the 6th–8th month (*p* < 0.05), followed by greater values at the 6th month than the 7th–11th month (*p* < 0.05) (Table 4).

The circulating ICa^2+^ of >10-year-old mares showed lower concentrations from the 1st to the 5th month of pregnancy than the values recorded at the 6th–11th month (*p* < 0.05) (Table 4).

Compared to > 10-year-old mares, those aged < 10 showed a significantly greater ratio of ICa^2+^/TCa^2+^Ca from the 5th to the 7th month of pregnancy (*p* < 0.05) (Figure 4).

Significant correlations between 5-HT and ICa^2+^ (r = 0.61; *p* < 0.05) and TCa^2+^ (r = 0.61; *p* < 0.05), between ICa^2+^ and TCa^2+^ (r = 0.81; *p* < 0.05), and between ICa^2+^/TCa^2+^ with ICa^2+^ (r = 0.57; *p* < 0.05) and TCa^2+^ Ca (r = −0.32; *p* < 0.05) were observed.

## 4. Discussion

The main goal of our study was to determine the possible interaction between 5-HT and calcemic status in healthy lactating pregnant mares to verify the existence of a 5-HT and Ca^2+^ axis. This work also evaluated whether the age of the mare could be associated with modifications in the concentrations of ICa^2+^ and TCa^2+^, as well as the ICa^2+^/TCa^2+^ ratio.

Unfortunately, not all the results obtained in this study were able to be compared with previous data in mares, so the data presented here could be considered a reference regarding the evolution of ICa^2+^, TCa^2+^, and the ICa^2+^/TCa^2+^ ratio concentrations throughout the entire pregnancy period.

The mean concentrations of ICa^2+^ in Purebred Spanish mares were like those previously described in adult horses of various breeds [25,26,27], although greater values have been reported by others [28,29]. In addition, the mean concentrations of TCa^2+^ were close to those reported in adult equines [26], although lower than those reported in foals [29].

Regarding the reproductive period, ICa^2+^ and TCa^2+^ in Spanish Purebred mares were similar, and the ICa^2+^/TCa^2+^ ratio was lower than those obtained in Thoroughbred and Quarter horse mares during the third trimester of gestation [18]. Compared to aged lactating pregnant mares, <10-year-old mares showed greater ICa^2+^ concentration throughout the entire pregnancy but lower TCa^2+^ concentration in the first 6 months of pregnancy, followed by greater values in the last 5 months of pregnancy.

However, the differences between results are complicated to evaluate, and this could be related to factors such as the analytical method, the type of sample or anticoagulant used, storage and collection methods, physical exercise, and nutrition, among others.

The results in mares are certainly different from those reported in women. In fact, although variable fluctuations with increases during pregnancy and decreases in the lactation phase have been reported in these mares, in women, ICa^2+^ concentrations remain constant within very narrow margins during gestation, although TCa^2+^ concentrations are considerably reduced in response to the expansion of extracellular fluids, the decrease in albumin concentrations (dilutional hypoalbuminemia of pregnancy), and the increase in the glomerular filtration rate, leading to an increase in calciuria [30,31,32].

ICa^2+^ represents the active circulating form that is physiologically maintained within a narrow range during pregnancy. It is mainly regulated by two hormones represented by the parathyroid hormone and calcitonin and by vitamin D_3_ and three effector organs (bone, kidney, and small intestine) by means of complex feedback loops [33]. Hence, its measurement provides a better status of circulating Ca^2+^ concentrations in lactating pregnant mares than assessing total Ca^2+^ ones.

Regression analysis shows strong positive correlations between ICa^2+^ and TCa^2+^, highlighting their impact at the physiological concentrations in lactating pregnant mares (r = 0.81). In addition, it is well known that the equine placenta, and specifically the areolar trophoblasts, is positive for the 9 KD Ca^2+^-binding protein, calbindin, involved in the transfer of Ca^2+^ from the mother to the fetus; what is more, the fetus is maintained in a hypercalcemic state, primarily by the activity of a placental Ca^2+^ pump located in the trophoblast’s basal plasma membrane [34]. Moreover, these differences in TCa^2+^ concentrations between mother and fetus do not persist during post-partum [18]. Since accelerated fetal skeletal development and mineralization are associated with increased Ca^2+^ demand in the third trimester, the physiological placental transfer of Ca^2+^ from the mare to the fetus may take into account these findings [34]; perhaps, this fact could explain why in the last period of gestation in the mare, the concentrations of ICa^2+^ and TCa^2+^ are reduced, coinciding with a period of maximum detailed growth.

In no case did the decrease both in ICa^2+^ and TCa^2+^ during the last two months of gestation reach hypercalcemic margins in the mares. However, in women, the fact that TCa^2+^ experiences a gradual decrease until the second trimester and a slight increase in the third has led to the postulation of the existence of a “physiological gestational hypocalcemia” [29]. In the same way, ionized hypocalcemia is a relatively common occurrence. Many potential causes of ionized hypocalcemia in association with pregnancy have been described, like nutritional and vitamin D_3_ deficiencies, malnutrition, hypoparathyroidism, and some chronic diseases [31,32].

During pregnancy and lactation, a situation of maternal hypocalcemia has been described, explained by the Ca^2+^ supply to the newborn through milk, which the mother would compensate for by reducing her Ca^2+^ urinary fractional excretion [35]. Although this type of hypocalcemia is very frequent in all dairy cow herds, where its prevalence reaches 78% [36] and there are clinical metabolic–nutritional pathologies caused by the decrease in blood Ca^2+^ [37], in no case could it be compared with those of the mares in this study. It would have been interesting to compare lactating mares with non-lactating mares, although this was not possible due to the production system in the farms collaborating for the realization of this work.

Regarding the effect of aging on ICa^2+^ and TCa^2+^, a previous study in horses showed no differences associated with age [38]. In contrast, the results obtained in this study show greater ICa^2+^ and TCa^2+^ concentrations and their ratio in younger mares between the second and third trimester of pregnancy. However, the interpretation of the results is made difficult by the impossibility of comparative data related to the equine species, where subjects are generally classified as adults or foals and not detailed by age classes. However, as organisms age, alterations and significant changes in mineral concentrations occur, all of which have pivotal implications for the physiological role of Ca^2+^. Additionally, increased attention has been paid to cellular senescence, showing that these natural and predictable phenomena are associated with aging and disturbed Ca^2+^ homeostasis [39]. This underscores the complexity of mineral interactions and their critical roles in biological processes like lactation, pregnancy, and aging.

Recently, an interesting relationship between 5-HT and Ca^2+^ has been demonstrated during lactation in dairy cows, with significant changes in both the parameters that physiologically occur at the onset of lactation and those that are mammary-driven, through a variety of direct and indirect 5-HT-stimulated mechanisms [8]. Although the 5-HT-Ca^2+^ axis is gaining clarity in dairy cows [8], understanding the physiological relevance in mares and its role in stimulating feedback mechanisms to fully integrate Ca^2+^ metabolism was the aim of this study. Different lactation strategies occur in a multiplicity of animal species so that the nutritional needs and requirements of the offspring can be provided. All of this can be accomplished through the mobilization of body stores to meet requirements for lipid, protein, and Ca^2+^ demands by the mammary gland for the synthesis of individual milk components [5,40]. Nevertheless, although different lactation strategies are dissimilar across animal species, all show an overlapping compromise in maternal physiology. On this basis, it is possible to presume that in lactating pregnant mares, the increase in nutrient demands by the mammary gland substantially changes the metabolic profile according to dam adaptations supporting their new physiological condition, as previously recorded in dairy cows [40,41,42].

Considering that in this study, as previously described, all mares were in lactation during the first 5 months of pregnancy with their delivered healthy foals, the metabolic demands of lactation are superimposed by the equally significant demands of pregnancy. What is more, in mares, the peak of lactation is achieved between 20 and 90 days according to different breeds [14]; hence, heavy breeds have a later peak than saddle horses. Therefore, it is possible to presume that the foals’ growth rate could affect milk yield, with greater production and a delayed peak in Spanish Purebred mares, characterized by an intense growth rate during the first months of life. In light of the above considerations, the evolution of 5-HT to decrease in the first trimester of pregnancy, coinciding with the peak of lactation, with a shift to increase from the 4th to the 6th month, is partially in accordance with the knowledge that dairy cows’ lactation results in a robust increase in circulating 5-HT concentrations [43], demonstrating that mammary-derived 5-HT significantly contributes to blood 5-HT during lactation [44]. Moreover, on the basis of this biphasic 5-HT profile (lower in the first trimester and greater in the second trimester), it is possible to also consider 5-HT a regulator of mammary homeostasis in lactating pregnant mares but with a lower percentage of circulating 5-HT being synthesized and secreted from mammary epithelial cells during lactation compared to ~50% recorded in dairy cows by Weaver et al. [44].

In addition, the consensual lower ICa^2+^ concentrations in the same first trimester confirm the previous results described by Harvey et al. [10] in mares in which the serum Ca^2+^ concentration was slightly decreased at two timeframes during lactation and in the first 2 months of gestation when mares were in lactation during those months compared to the values of mid-gestation. Moreover, the evolution of an increase from the 3rd to the 6th month confirms that the ICa^2+^ aliquot is more useful than TCa^2+^ and provides a better indication of being calcemic since ICa^2+^ is biologically active and is closely regulated by Ca^2+^-modulating hormones, as previously described [8] and corroborated by the existence of the positive correlation between them.

Moreover, the 5-HT decrease during the first trimester, described both in mares younger and older than 10 years, could be an expression of a defensive strategy of the mother, with the implementation of negative feedback on 5-HT; in fact, in this timeframe, it is secreted not only by the mammary gland but probably also by the trophoblast cells of the feto-placental unit, as described in women [45]. Moreover, in women, 5-HT receptors are broadly expressed in female reproductive tissues [45], and the placenta represents both the primary source of 5-HT and the regulator of its fetal forebrain concentration, which is synthesized from maternal tryptophan [46,47]. In addition, 5-HT transporters at the trophoblast cell membranes regulate the early embryonic blood flow via placental vascular beds, and 5-HT regulates both trophoblast proliferation and viability [48,49]. Although detailed data and studies do not exist in the equine species as in women, considering similarities in their reproductive physiology [50,51], some extrapolation could also be made for 5-HT underlying the importance of pregnancy and placental mechanisms in driving its crucial synthesis during development.

These results confirm that while the manipulation of both Ca^2+^ and 5-HT metabolisms during this timeframe resulted in related positive effects on the ensuing lactation [52,53,54], on the one hand, a diet rich in tryptophan, a precursor of 5-HT, induces negative effects on fetal growth hormone secretion, inducing its limited development and growth [55] while also increasing the risk of miscarriage [21]. The significant increase in circulating 5-HT concentrations in Spanish Purebred mares from the 4th to the 6th month of pregnancy was in line with the data recorded in women [56], with peaks in the second and third trimesters. One of the reasons for this rise during pregnancy is the pivotal importance of 5-HT hormones and neuromodulators, contributing to glucose homeostasis and adiposity observed in women [57], mice, and sheep [58]. Although a temporal increase in circulating 5-HT concentrations is common in women during healthy and successful pregnancies, greater increases in their concentration cause inevitable harmful effects [59].

Hence, the successive decrease in 5-HT from the 7th to the 11th month could be due to maternal physiological mechanisms, Ca^2+^ metabolism, and fetal-derived endocrine signals that replace mammary-derived endocrine signals that are now extinct. These results suggest the existence of a coordinated 5-HT-Ca^2+^ feedback loop involving endocrine and autocrine/paracrine mechanisms that modulate and guarantee maternal and mammary Ca^2+^ homeostasis.

Balancing calcemic requirements during lactation comes at a maternal cost, one that is aided by the mammary gland through the mammary-derived endocrine signals, PTHrP (parathyroid hormone-related protein), and 5-HT [5,6].

A field of study on serotonin’s influence on Ca^2+^ homeostasis during lactation both in rodents and dairy cows [41,53,59,60] has gained recent attention, and an additional scientific segment could also extend knowledge about this subject in mares. Consideration of such information is important in recognizing the existence of the 5-HT-Ca^2+^ axis in healthy lactating pregnant mares to assess its potential effects on the reproductive physiology of equine species. This suggests that the 5-HT-Ca^2+^ axis induces not a random chance event but an interactive process between maternal and fetal endocrine and metabolic homeostasis.

## 5. Conclusions

The data obtained show that the pregnancy and lactation phases of mares are associated with 5-HT changes according to different ages and the progression of aging. We believe that, together, these results provide a comprehensive and updated review of progress in a dynamic 5-HT-Ca^2+^ axis in healthy lactating pregnant mares. The better use of animal models, more advanced knowledge of the molecular environment of 5-HT receptors, and, finally, deeper knowledge of the interaction among 5-HT neurotransmission and other biological systems could represent future frontiers.

## Figures and Tables

**Table 1:** Circulating serotonin (5-HT) concentrations (Mean ± S.D.) in lactating pregnant Spanish Purebred mares aged < 10- and >10 years old throughout the entire pregnancy.

Month of Pregnancy	5-HT (ng/mL) (<10 Years Old)	5-HT (ng/mL) (>10 Years Old)
1	430.4 ± 41.4 ^a^	352.9 ± 76.4 ^A^
2	450.0 ± 58.8 ^a^	249.3 ± 85.3 ^A^
3	358.6 ± 26.4 ^a^	211.1 ± 106.9 ^A^
4	475.5 ± 76.9	259.9 ± 20.9
5	598.1 ± 141.1	333.6 ± 203.8
6	709.7 ± 57.1 ^b^	639.7 ± 81.5 ^B^
7	591.9 ± 145.9	482.7 ± 63.2
8	673.2 ± 92.8	645.9 ± 85.2
9	591.8 ± 119.3	444.6 ± 87.7
10	456.6 ± 207.1	446.1 ± 139.6
11	205.4 ± 11.1 ^c^	215.0 ± 23.5 ^C^

Letters indicate significant differences (*p* < 0.05) vs. other months. ^a^ = vs. 5–10 months; ^b^ = vs. 4–5 months; ^c^ = vs. all previous months. ^A^ = vs. 5–10 months; ^B^ = vs. 4–5 months; ^C^ = vs. all previous months.

**Table 2:** Circulating ionized Ca^2^ (ICa^2+^) concentrations (Mean ± S.D.) in lactating pregnant Spanish Purebred mares aged < 10- and >10 years old throughout the entire pregnancy.

Month of Pregnancy	ICa^2+^ (mmol/L) (<10 Years Old)	ICa^2+^ (mmol/L) (>10 Years Old)
1	1.46 ± 0.07 ^a^	1.41 ± 0.14 ^A^
2	1.38 ± 0.01 ^a^	1.35 ± 0.02 ^A^
3	1.47 ± 0.05 ^a^	1.40 ± 0.05 ^A^
4	1.58 ± 0.01 ^a^	1.42 ± 0.10 ^A^
5	2.19 ± 0.44 ^b^	1.53 ± 0.10
6	2.47 ± 0.69 ^b^	1.69 ± 0.17
7	2.15 ± 0.22	1.83 ± 0.29
8	1.95 ± 0.22	1.67 ± 0.21
9	1.98 ± 0.28	1.72 ± 0.40
10	1.70 ± 0.07	1.52 ± 0.15
11	1.55 ± 0.05 ^a^	1.44 ± 0.11 ^A^

Letters indicate significant differences (*p* < 0.05) vs. other months. ^a^ = vs. 5–9 months; ^b^ = vs. 8–11 months. ^A^ = vs. 7–9 months.

**Table 3:** Circulating total Ca^2+^ (TCa^2+^) concentrations (Mean ± S.D.) in lactating pregnant Spanish Purebred mares aged < 10- and >10 years old throughout the entire pregnancy.

Month of Pregnancy	TCa^2+^ (mmol/L) (<10 Years Old)	TCa^2+^ (mmol/L) (>10 Years Old)
1	2.42 ± 0.06 ^a^	2.58 ± 0.03 ^A^
2	2.53 ± 0.09 ^a^	2.62 ± 0.03 ^A^
3	2.62 ± 0.11 ^a^	2.76 ± 0.07 ^A^
4	2.81 ± 0.24 ^b^	2.83 ± 0.08 ^B^
5	2.88 ± 0.13 ^b^	2.86 ± 0.09 ^B^
6	2.55 ± 0.32	2.58 ± 0.15
7	2.62 ± 0.23	2.31 ± 0.14
8	2.53 ± 0.17	2.23 ± 0.17
9	2.74 ± 0.15 ^c^	2.65 ± 0.1 ^C^
10	2.38 ± 0.13	2.16 ± 0.13
11	2.20 ± 0.15	2.05 ± 0.09

Letters indicate significant differences (*p* < 0.05) vs. other months. ^a^ = vs. 3–5 months; ^b^ = vs. 6–11 months: ^c^ = vs. 10–11 months. ^A^ = vs. 3–5 months: ^B^ = vs. 6–11 months: ^C^ = vs. 10–11 months.

**Table 4:** Ratio of total Ca^2+^/ionized Ca^2+^ (ICa^2+^/TCa^2+^) (Mean ± S.D.) in lactating pregnant Spanish Purebred mares aged < 10- and >10 years old throughout the entire pregnancy.

Month of Pregnancy	ICa^2+^/TCa^2+^ Ratio (<10 Years Old)	ICa^2+^/TCa^2+^ Ratio (>10 Years Old)
1	060 ± 0.03 ^a^	0.54 ± 0.05 ^A^
2	0.54 ± 0.02 ^b^	0.52 ± 0.01 ^A^
3	0.56 ± 0.02 ^b^	0.51 ± 0.01 ^A^
4	0.56 ± 0.04 ^b^	0.50 ± 0.03 ^A^
5	0.76 ± 0.14 ^c^	0.53 ± 0.03 ^A^
6	0.93 ± 0.15 ^d^	0.65 ± 0.03
7	0.80 ± 0.08	0.78 ± 0.09
8	0.77 ± 0.10	0.75 ± 0.07
9	0.72 ± 0.07	0.65 ± 0.17
10	0.71 ± 0.05	0.70 ± 0.05
11	0.70 ± 0.04	0.70 ± 0.03

Letters indicate significant differences (*p* < 0.05) vs. other months. ^a^ = vs. 5–9 months; ^b^ = vs. 5–11 months; ^c^ = vs. 6–8 months; ^d^ = vs. 7–11 months. ^A^ = vs. 6–11 months.

## Data Availability

The data that support this study will be shared upon reasonable request to be corresponding author.
